# Tying of the Carotid Artery, Followed by Softening of the Brain, and Death

**Published:** 1852-02

**Authors:** 


*Tying of the Carotid Artery, followed by Softening of the Brain, and Death.*—The carotid artery has now been tied several times, with perfect success, both in this country and on the continent; but when deligation of that artery for aneurism has been followed by sudden softening of the brain and a fatal issue, the circumstance should always be recorded. Dr. Chopel, a hospital surgeon of St. Malo, in France, lately brought such a case before the Academy of Medicine of Paris. From pressure of the aneurismal tumor, severe pain was experienced by the patient, and Dr. Chopel preferred the ligature to electro-puncture, which latter operation he deprecates very warmly. The author suspects that a tendency to softening or complete *ramollissement* existed before the operation, although no striking symptoms of such pathological change existed. The patient died a few days after the deligation of the artery, with all the symptoms of softening of the brain.—*Lancet.*

